# A Case of Displacement of the Heart, in Consequence of Chronic Disease of the Chest

**Published:** 1853-09

**Authors:** W. C. Sneed

**Affiliations:** Secretary Kentucky State Medical Society, Frankfort, Kentucky


Art. II.—*A Case of Displacement of the Heart, in Consequence of Chronic Disease of the Chest.* By W. C. Sneed, M.D., Secretary Kentucky State Medical Society, Frankfort, Kentucky.

Displacement of the heart, from any cause whatever, so seldom occurs that few practitioners of medicine out of the large hospitals ever meet with an instance. The following case is the only one that has ever fallen under my notice, and as it presented some striking peculiarities, I feel disposed to present a history of it to the profession through the medium of the Western Journal.

The subject was a negro girl, about twelve or thirteen years old, a history of whose case, as near as I can obtain it, is as follows: About two years since she had an attack of pleurisy, or pleuro-pnenmonia, while living in the family of a physician of this place, since deceased, who, I presume, treated her not only promptly and skilfully but with all the kindness and care that the nature of her case required. The girl was returned to her owner some time during the year in bad health, with a cough, considerable emaciation, and laboring under other symptoms which clearly indicated chronic pleurisy. After her return to her master, she was placed under the medical care of two other physicians, who attended her for some time with evident manifestations of improvement, but not with permanent relief. I have no details of the treatment she received from any of the medical gentlemen who had charge of the case in its earlier stages, but have no doubt the treatment she received was the most appropriate to her case.

My partner, Dr. Rodman, was called to see the case in August, 1852, and upon examination found the heart pulsatory in the right side immediately opposite to, but farther over than, its normal position in the left.

The left lung was unusually resonant, and its capacity greatly increased by the diseased condition of the right. There was dullness throughout the right side on percussion, and the healthy murmurs were all absent; in fact, the lung was entirely or nearly impervious.

The heart's action was rapid, full, and regular, and the breathing more than doubled in frequency. The patient was feeble, emacited, and had hectic fevers, with occasional night sweats and some diarrhea.

In a conversation upon the subject of this case, held with the medical gentlemen who had preceded him in the treatment of the case, they expressed surprise at the announcement of such a condition of the heart; but, upon a thorough examination of the patient in our presence, they discovered the true state of the case. The patient continued feeble and emaciated, suffering with a troublesome cough, hectic fever, with rapid action of the heart, until the latter part of July, 1853, when she expired.

I was permitted to make a post mortem examination, which was done in the presence and with the aid and assistance of my partner, Dr. Rodman, and my friend, Dr. J. M. Mills. I am indebted to Dr. Mills for the following details of the post mortem appearances.

*Autopsy.*—The right side of the thorax was larger, more rounded, and the ribs more elevated, than the left. Upon removing the sternum, the pericardium was found distended with serum, and the heart occupying a position on the right side of the body, two inches further over than the normal position it should have occupied on the left. In fact, it presented the appearance of having been pushed directly across to the spot where it was found. Its anterior surface was flattened, the walls of the left ventricle thickened, and a cartilaginous ring around the internal surface of the aorta at its commencement.

The left lung was filled with tubercles, and its anterior border extended a couple of inches beyond the median line into the right side of the cavity. The right lung was adherent, throughout its entire extent of surface, to the walls of the chest, but was so exceedingly small and contracted as to be almost concealed when grasped by the hand. The lower third entirely absorbed.

The abdominal viscera were healthy in appearance, except a slight enlargement of the liver.

I may here state that the post-mortem appearances were in almost exact accordance with the diagnosis made when first seen by Dr. Rodman and myself, which diagnosis was concurred by the two physicians who preceded us in the treatment of the case. Our conclusions were about as follows:

1st. That the patient had, in the first place, pleuro-pneumonia, the inflammation extending to the whole pleural surface, which was followed by extensive adhesions of the pleura, gluing the right lung to the mediastinum, and all the subjacent tissues.

2nd. Atrophy of the substance of the right lung with contrraction of the right side, and a consequent pulling of the heart, pericardium and mediastinum with it.

Owing to the loss of the right lung there was increased capacity added to the left, and a pushing over of the heart and its appendages by the left lung.

The patient's life and sufferings were terminated by the deposit of tubercular matter in the left lung, it being filled with tubercular deposits in a partially softened state when the lung was examined.

Displacements of the heart, to a trifling extent, are produced by a variety of causes, the more common of which is an effusion of air or liquid into one of the pleural cavities. The displacement is most manifest when the effusion is in the left side, the heart being then pushed over into the right, the degree of displacement depending on the amount of effusion; a condition which always exists in cases of empyema, hydrothorax and pneumothorax. As a general rule, the more rapid the effusion the more certainly will the displacement occur, and the greater will be the extent.

In a great majority of cases of removal of the heart out of its natural situation, the cause is either empyema or pneumothorax. I have met with several such cases, but the one here reported is the first met with produced by contraction of the right lung. In chronic dropsical effusions into the chest, displacement of the heart is not so apt to follow, because the effusion is slow and gradual, and the extensibility of the neighboring textures is more completely brought into play. If there is displacement at all it is generally slight. When the effusion occurs on the right side, the heart may be pushed more to the left and upwards than is natural, but to effect this a considerable effusion is necessary. In a case reported by Dr. Townsend, of England, the heart was distinctly seen and felt pulsating between the fourth and fifth ribs, near the left axilla, after paracentisis; the heart gradually returned to its normal position as the displacing force was removed by drawing off the air and fluid contained in the opposite pleura.

Dr. Stokes ascertained, by observing some cases which fell under his notice, that the absorption of an effusion of the right side would cause the heart to be displaced to that side, the pleural cavity being obliterated by lymph, while the lung of the left side was enlarged, so as to aid in occupying the vacant space, and pushing the heart over.

Tumors existing in the left sac of the pleura may lead to this displacement; aneurismal tumors may push the heart to the right, to the left, and upwards, or even forwards, and outwards, against the walls of the thorax, or downwards, so that its apex will pulsate in the epigastrium. Dr. Townsend relates a case of the latter character. In a case recorded by Drs. Graves and Stokes, (Dublin Hospital Reports, vol. V.. p. 19,) the heart was pushed upwards and to the right side by an abdominal aneurism, so as to pulsate in the intercostal space of the third and fourth ribs. Dr. Hope mentions the displacement to the left by an aneurism of the ascending aorta. He further remarks that any cause which pushes the diaphragm upwards, and prevents its descent, such as distention of the abdomen by an enlarged viscus, a tumor, or an effusion, will change the position of the heart, so that its axis will be directed horizontally; and further, that the same position may be produced by an adhesion of the pericardium to the heart, by which its enlargement is prevented. Hernia of the diaphragm is another frequent cause of displacement of the heart. In a case reported by Drs. Graves and Stokes, the stomach and a large portion of the transverse arch of the colon were lodged in the left cavity of the thorax, and pushed the heart and mediastinum towards the right side. Enlargement of the air cells of the lungs will produce displacement of the heart; the enlarged lung will push the diaphragm downwards, thus increasing the vertical diameter of the chest, or it may suffer a slight degree of displacement laterally, by the mediastinum being pushed to the right side of the lung.

The most remarkable case on record is that reported by Dr. Stokes, which he terms "dislocation" of the heart, and was produced by external violence. The patient was crushed between a water-wheel and the embankment on which the axle was supported. Several ribs were broken, as well as the right clavicle and humerus. The heart, which, according to the statement of the patient, had always occupied its natural situation, was found beating at the right side, (Med. Gazette, Vol. VIII.)

AUGUST, 1853.

